# A Novel Inhalable Dry Powder to Trigger Delivery of Voriconazole for Effective Management of Pulmonary Aspergillosis

**DOI:** 10.3390/pharmaceutics16070897

**Published:** 2024-07-04

**Authors:** Alanood S. Almurshedi, Sarah N. Almarshad, Sarah I. Bukhari, Basmah N. Aldosari, Samiah A. Alhabardi, Fai A. Alkathiri, Imran Saleem, Noura S. Aldosar, Randa Mohammed Zaki

**Affiliations:** 1 Department of Pharmaceutics, College of Pharmacy, King Saud University, P.O. Box 2457, Riyadh 11451, Saudi Arabia; salmarshadi@ksu.edu.sa (S.N.A.); sbukhari@ksu.edu.sa (S.I.B.); baldosari@ksu.edu.sa (B.N.A.); salhabardi@ksu.edu.sa (S.A.A.); falkathire@ksu.edu.sa (F.A.A.); 2 School of Pharmacy & Biomolecular Sciences, Liverpool John Moores University, Liverpool L3 3AF, UK; 3 Department of Botany and Microbiology, College of Science, King Saud University, P.O. Box 2457, Riyadh 11451, Saudi Arabia; 4 Department of Pharmaceutics, College of Pharmacy, Prince Sattam Bin Abdulaziz University, P.O. Box 173, Al-Kharj 11942, Saudi Arabia; r.abdelrahman@psau.edu.sa; 5 Department of Pharmaceutics and Industrial Pharmacy, Faculty of Pharmacy, Beni-Suef University, P.O. Box 62514, Beni-Suef 62514, Egypt

**Keywords:** voriconazole, invasive pulmonary aspergillosis, dry-powder inhaler, nanoliposomes, L-leucine, anti-fungal activity, cell viability, loading entrapment

## Abstract

Invasive pulmonary aspergillosis (IPA) is a fatal fungal infection with a high mortality rate. Voriconazole (VCZ) is considered a first-line therapy for IPA and shows efficacy in patients for whom other antifungal treatments have been unsuccessful. The objective of this study was to develop a high-potency VCZ-loaded liposomal system in the form of a dry-powder inhaler (DPI) using the spray-drying technique to convert liposomes into a nanocomposite microparticle (NCMP) DPI, formulated using a thin-film hydration technique. The physicochemical properties, including size, morphology, entrapment efficiency, and loading efficiency, of the formulated liposomes were evaluated. The NCMPs were then examined to determine their drug content, production yield, and aerodynamic size. The L3NCMP was formulated using a 1:1 lipid/L-leucine ratio and was selected for in vitro studies of cell viability, antifungal activity, and stability. These formulated inhalable particles offer a promising approach to the effective management of IPA.

## 1. Introduction

Fungal infections affect over 150 million people globally, with a mortality rate of 1.6 million [1]. Pulmonary aspergillosis, caused by Aspergillus spp., is one of the most common fungal infections affecting the lungs, with over 14 million cases worldwide. It can lead to severe bronchopulmonary syndromes such as allergic bronchopulmonary aspergillosis, chronic pulmonary aspergillosis, and invasive pulmonary aspergillosis (IPA) [2]. IPA is caused by inhaling airborne fungal spores, leading to respiratory and constitutional symptoms. Due to the rapid progression of the disease and its high mortality and morbidity rates, treatment should be initiated immediately [3].

Voriconazole (VCZ), a second-generation antifungal drug, is the primary treatment for IPA. The systemic administration of VCZ has limitations, such as high inter- and intra-patient pharmacokinetic variability, poor lung distribution, and systemic toxicity. Additionally, the plasma levels of P450 isoenzymes can vary inter-individually due to factors such as liver function and polymorphisms. [4]. Moreover, VCZ has poor water solubility, which could reduce its efficiency [5]. An alternative strategy used to circumvent this issue is to formulate VCZ within nanocarriers that target macrophages where infections reside. Pharmaceutical nanocarriers are submicron-sized drug delivery vehicles with high levels of versatility; this category includes polymeric, lipidic, and inorganic nanoparticles; liposomes; nanocomplexes; niosomes, and many others. [6]. The most important characteristics of nanocarriers are particle size, shape, and dispersity (heterogeneity of particles in terms of size, as expressed by the polydispersity index (PDI)). The particle size and shape affect the biodistribution and elimination of nanocarriers [7]. They also affect their attachment, firm adhesion, cellular distribution, circulation half-life, and cellular uptake [8].

Liposomes are spherical lipid vesicles comprised of amphiphilic phospholipids that assemble into one or more lipid bilayers surrounding an aqueous core. Due to their structure, liposomes can encapsulate both hydrophobic and hydrophilic compounds. They have been used in various therapeutic nanoparticle formulations to reduce host toxicity and improve the solubility of drugs for use in applications [9]. The main objectives of a method for liposome nanoformulation formation are to produce uniform particles with a narrow size-distribution, achieve the required degree of lamellarity, ensure efficient drug encapsulation, and maintain long-term colloidal stability of the products. However, controlling their size distribution can be challenging. Characterizing NPs after preparation is essential to ensuring their suitability for in vitro and in vivo applications [7]. When characterizing the particle size distribution of liposomes, we use a parameter called the “polydispersity index” (PDI). The term “polydispersity” (or “dispersity”, as recommended by IUPAC) is used to describe the degree of non-uniformity of a size distribution of particles [10]. Also known as the heterogeneity index, PDI is a number calculated from a two-parameter fit to the correlation data (the cumulants analysis). This index is dimensionless, and the numerical value of PDI ranges from 0.0 (for a perfectly uniform sample with respect to the particle size) to 1.0 (for a highly polydisperse sample with multiple particle-size populations). In practice, values of 0.2 and below are most commonly deemed acceptable for NP materials [11].

Recent studies have highlighted the advantages of using liposomes for pulmonary drug delivery. Liposomal drug delivery systems present an appealing option for targeted drug delivery to the lungs through inhalation. This system offers controlled-release properties that extend the drug’s therapeutic effects over an extended duration. In comparison to free drugs, liposomes improve drug delivery and deposition in the lungs, enabling a more effective targeting of specific lung sites. This may reduce dosing frequency and decrease the pulmonary irritation associated with VCZ [12]. The use of liposomal carrier systems in inhalation applications can reduce drug toxicity, enhance drug stability, and minimize pulmonary clearance [13]. Furthermore, liposomal encapsulation has demonstrated a reduction in systemic circulation entry, leading to improved drug distribution within the lung airspace. This approach has the potential to decrease the frequency of drug dosing, thereby enhancing patients’ quality of life and reducing healthcare costs [14]. Significantly, liposomes can facilitate the targeting of specific pulmonary cells, such as macrophages that harbor bacteria. This is because liposomes can fuse to the fungal cell membrane and facilitate the transference of VCZ, in an internalization mechanism that has already been elucidated using AmBisomeVR [15]. One practical approach to increasing the antifungal effect is to achieve a high local drug concentration via localized drug delivery, such as pulmonary administration.

Pulmonary drug delivery systems (PDDSs) are gaining popularity as one of the safest, quickest, and most efficient methods for administering medications for both local and systemic treatment. A significant advantage of inhaled therapy is the ability to achieve higher concentrations at the site of action than with oral or intravenous doses. Furthermore, PDDSs deliver drugs directly to the disease site, reducing systemic exposure and toxicity. This allows for the direct deposition of high drug concentrations at the site of infection, via the alveolar epithelium, and absorption into the bloodstream, using a distinctive delivery device [16]. Various conventional pulmonary delivery systems are available, including dry-powder inhalers (DPIs), pressurized metered-dose inhalers, soft-mist inhalers, and nebulizers [17,18,19]. The pulmonary delivery of high doses, specifically of antibiotics, antifungals, and antivirals, is only possible using nebulizers and DPIs. However, nebulizers have several drawbacks, which are primarily related to patients’ compliance. In comparison, dry-powder inhalers (DPIs) are an effective and affordable method of delivering high doses of medication to the lungs. DPIs are portable and easy to use and require less maintenance than do liquid nebulizers. They also have a short operation time, making them a convenient option for patients. Moreover, delivery to the lungs is induced by inspiratory flow and does not require a compressor. Due to these benefits, DPIs can improve patients’ compliance with their prescribed treatment plan [20]. Additionally, DPI formulations are essential for stabilizing the active ingredients in medication, which helps maintain their potency and ensure their effectiveness. This is especially important for antifungal drugs used to treat Aspergillus infections, as they must reach small airways to be effective [21]. The effectiveness of DPIs, however, hinges on various factors, including the physicochemical properties of the drug formulation (moisture sensitivity), the design and functionality of the inhaler device, and the patient’s inhalation technique [22]. Achieving consistent drug delivery to the lungs remains a challenge in the development of DPIs. Ongoing research and innovation are needed to optimize aerosol generation and particle dispersion upon inhalation. Advances in device design and formulation development have been instrumental in overcoming these challenges, enhancing the efficiency and therapeutic performance of DPIs [23]. A drying step is frequently needed to produce dry powders for inhalation. Spray drying (SD) is the most widely used technique to produce liposomal dry-powder inhalers. It has the benefit of generating particles with well-regulated and repeatable physicochemical features. Additionally, DPI formulations play a crucial role in stabilizing the active ingredients of medication, which helps to preserve their potency and ensure their efficacy. By maintaining the quality of the active ingredients, DPI formulations help to ensure that the medication is as effective as possible, providing the best possible treatment for the intended condition [24,25]. Antifungal drugs must reach small airways to treat Aspergillus infections. For DPI formulations to be effective, the drug particles need to be between 1 and 5 µm in size. This size range enables the particles to reach and treat small airways effectively [26]. However, when delivering NPs to the lungs, there is a high likelihood of exhalation before the particles can be deposited. Additionally, NPs tend to aggregate strongly upon aerosolization under normal airflow rates in passive DPIs, and their cohesive nature makes handling them extremely difficult. To overcome the limitations of nanoparticles, researchers have proposed the use of nanocomposite microparticles (NCMP), which involves controlled agglomeration of NPs into micron-sized clusters. This approach harmonizes the advantages of nanoparticles with the aerodynamics of small microparticles. Therefore, nanocomposite microparticles (NCMP) can achieve improved bioavailability and improved aerosolization behavior of the drug. Therefore, SD of nanoliposomes to inhalable NCMPs of 1–5 μm can be used to effectively administer NPs to the lungs using DPIs.

Therefore, formulating a liposomal VCZ DPI formulation via SD for local pulmonary delivery may offer advantages over the current long-term oral VCZ treatment regime in targeting the site of action and minimizing possible systemic side effects. This may result in improved clinical approaches for patients with chronic respiratory infections.

## 2. Materials and Methods

### 2.1. Materials

VCZ (99.8% purity) was purchased (from Selleck Chemicals, Houston, TX, USA), and 1,2-dioleoyl-sn-glycero-3-phosphocholine (DOPC), 1,2-distearoyl-sn-glycero-3-phosphocholine (DSPC), and 1,2-dioleoyl-sn-glycero-3-phosphoethanolamine (DOPE) were provided by Lipoid (Steinhausen, Switzerland). HEPES (4-(2-hydroxyethyl)-1-piperazineethanesulfonic acid), phosphate-buffered saline (PBS) powder (pH 7.4), triethylamine (TEA), sodium hydroxide pellets, Tween 80, and L-leucine (LEU) were purchased from Sigma–Aldrich (Saint Louis, MO, USA). A495 cells were obtained from the American Type Culture Collection (ATCC, Manassas, VA, USA). The cells were maintained at 37 °C and 5% CO2 in RPMI 1640 medium (GIBCO^®^ Waltham, MA, USA) containing 10% fetal bovine serum (FBS) and 1% antibiotic/antimycotic, which were purchased from GIBCO^®^, InvitrogenTM, Carlsbad, CA, USA. All other reagents and chemicals were of analytical grade. The fungal strains *Aspergillus niger* ATCC 1023 and *Aspergillus flavus* were provided by the Central Laboratory, College of Pharmacy, King Saud University.

### 2.2. Methods

#### 2.2.1. Preparation of VCZ-Loaded Liposomes (VCZ-Ls)

The liposomal formulations were prepared via thin-film hydration followed by extrusion [7]. In a round-bottomed flask, VCZ and all of the lipids (DSPC, DOPC, and DOPE in a 3:10:3 molar ratio) were dissolved in a solvent mixture of chloroform and methanol (2:1, *v*/*v*). VCZ was added to the total lipids in the following VCZ/lipid molar ratios: F 1 (0.5:1), F 2 (1:1), and F 3 (1.5:1). Then, the solvent was evaporated using a rotary evaporator (Buchi Rotavapor R-200, Switzerland) at 60 °C and 100 rpm under reduced pressure to obtain a thin layer on the inner side of the round-bottomed flask. The lipid film was hydrated with HEPES-buffered saline (pH 7.4) for 30 min in a bath sonicator at 60 °C. The liposomal dispersion was subjected to extrusion to create smaller vesicles using a syringe extruder (Liposo-Fast™, Ottawa, ON, Canada). Finally, the resulting products were stored in a refrigerator at 4 °C overnight before characterization.

#### 2.2.2. Physicochemical Characterization of the VCZ-Ls

##### Particle Size, Polydispersity Index, and Zeta-Potential Measurements

Dynamic light scattering (DLS) was conducted using a Zetasizer (Nano ZS; Malvern, UK) to measure the particle size, PDI, and zeta potential of the VCZ-Ls. Before measurements were taken, the liposomes were diluted with distilled water as required. Particle size analysis was performed using disposable cuvettes, and zeta-potential measurements were carried out in clear zeta dip cells. All samples were measured in triplicate at 25 °C.

##### Morphological Analysis

The VCZ-Ls formulations were analyzed to determine their morphology using transmission electron microscopy (TEM) (JEM-1400 electron microscope from JEOL, Tokyo, Japan). For this purpose, a diluted liposomal formulation was dropped onto a formvar film grid. After excess liquid was removed with filter paper, the formulation was air-dried for 15 min and observed via TEM.

##### Encapsulation Efficiency

The ultracentrifugation technique was used to evaluate the EE % of the VCZ-Ls. The liposomes were centrifuged at 30,000 rpm for 40 min at 4 °C (Sigma-Aldrich, Darmstadt, Germany). The supernatant containing the free dissolved VCZ was collected and analyzed via HPLC to quantify the unentrapped drug. All samples were filtered using a 0.45 μm syringe filter before being analyzed. The amount of VCZ was quantified using High-Performance Liquid Chromatography (HPLC), based on a previously described method [27]. The process was repeated three times for each sample. The EE% was estimated using the following equation [28]:

(1) $$EE\%=\frac{\left(Total\;amount\;of\;VCZ-Free\;amount\;of\;VCZ\right)}{Total\;amount\;of\;VCZ}\times 100\;$$

##### In Vitro Release Study

The in vitro release profile of VCZ from the VCZ-Ls was evaluated using the Franz diffusion cell system (FDC-6, LOGAN, Instruments Corporation, San Diego, CA, USA) [29]. Cellophane cellulose dialysis membranes (molecular weight cut-off: 12–14 KDa) were kept in distilled water overnight at room temperature for 12 h prior to use to ensure wetting. Then, the membranes were mounted between the diffusion cells’ donor and receptor compartments. An aliquot of liposomes or VCZ suspension was added into donor chambers, ensuring that no air bubbles were present under the membrane. The receptor compartment consisted of 5 mL of PBS buffer, pH 7.4, with 0.2% Tween 80 to maintain sink conditions, at 37 °C; stirring was maintained at 150 rpm. Samples of 0.5 mL were withdrawn at intervals up to the 8 h mark, and replaced immediately with an equal volume of fresh buffer to maintain sink conditions. All samples were filtered using a 0.45 μm syringe filter before being analyzed via HPLC [27]. The experiments were performed in triplicate. The kinetic parameters of the obtained release data were fitted to different kinetic models, including zero-order, first-order, Higuchi, Korsmeyer–Peppas, Peppas–Sahlin, and Hixson–Crowell models, to estimate the mechanism of drug release from VCZ-Ls, using DDSolver software version 3.2 [30].

#### 2.2.3. Preparation of Nanocomposite Microparticles (NCMPs)

SD was used to prepare NCMPs with different LEU ratios. The optimized formulation of VCZ-Ls was dispersed in LEU solution at different lipid/LEU ratios (1:0.5 (L1NCMP), 1:1 (L2NCMP), and 1:1.5 (L3NCMP)); mixed for 5 min at 25 °C; and spray-dried at a feed rate of 10%, an aspirator capacity of 85%, an atomizing air flow of 500 L/h, an inlet temperature of 100 °C, and an outlet temperature of ~50–55 °C. A Büchi B-290 mini spray-dryer (Büchi Labortechnik, Flawil, Switzerland) with a nozzle atomizer diameter of 0.7 mm and a high-performance cyclone (Büchi Labortechnik) were used to separate the dry powders (NCMPs) from the air stream. The NCMPs were collected and stored in a desiccator pending further analysis.

#### 2.2.4. Characterization of Nanocomposite Microparticles (NCMPs)

##### Spray-Dried Powder Yield (%)

The powder yield was determined as a percentage of the original mass of the powder used, adopting the following equation [31]:

(2) $$Yield\;\%=\frac{Weight\;of\;powder\;collected\;after\;spray\;drying}{Weight\;of\;total\;dry\;mass\;used\;for\;the\;preparation}\times 100$$

##### Determination of the Drug Content of the Spray-Dried Powders

The spray-dried powders (5 mg) were measured accurately and combined with one milliliter of an aqueous solution. The mixture was thoroughly stirred until the powders dispersed; at this point, 4 mL of ACN was introduced to the volumetric flask, bringing the total volume to 5 mL. The solution was then vigorously vortexed and filtered through a 0.22 μm membrane filter. Finally, the VCZ content was determined using HPLC [27].

##### Size and Morphology of Spray-Dried Powders

SEM was employed to determine the surface morphology of the powders (JSM-6060LV, JEOL Scanning Electron Microscope). Thin layers of the produced spray-dried powders were fixed onto stubs using carbon adhesive tape to obtain photographs of them. The samples were sputter-coated with platinum under an argon atmosphere at 180 mA for approximately one minute using an auto fine coater. Finally, the stubs were randomly scanned to capture the images.

##### Fourier Transform Infrared (FTIR) Spectroscopy

The structures of the NPs were analyzed using FTIR spectroscopy (Perkin Elmer FT-IR Spectrum BX apparatus, Perkin Elmer, Waltham, MA, USA). The FTIR spectra of the free VCZ powder, L3NCMP powder, and free LEU powder were prepared using the conventional potassium bromide (KBr) disc method. This method involved using 2 mg of the sample in 98 mg of KBr and examining it in transmission mode. The KBr disc was prepared at a pressure of 10 tons. The IR wavelength was between 400 and 4000 cm^−1^ in the mid-infrared region, with a resolution of 4 cm^−1^ determined using the KBr Pellet method. The recorded spectra give the positions of bands in relation to the nature and strength of the bonds and specific functional groups, thus providing information about molecular structures and interactions.

##### Cell Viability Study

An MTT assay (Promega, San Luis Obispo, CA, USA) was used to evaluate the cell viability profiles of VCZ, Blank LNCMPS, and L3NCMPs. A549 cell lines were seeded into 96-well plates at a density of 1 × 10^4^ cells per well and incubated overnight in a complete culture medium. Then, different concentrations of VCZ (1 µM to 25 µM) were added to each well and incubated for an additional 72 h. Cell viability was then determined using an MTT assay. Briefly, the cells were washed with PBS solution and incubated with 0.2 mL fresh Dulbecco’s Modified Eagle Medium (DMEM) containing 0.5 mg/mL MTT (Sigma, Saint Louis, MO, USA) for 3 h. The medium was removed, and MTT formazan was dissolved in 0.2 mL dimethyl sulfoxide (DMSO). The optical density was then determined at 550 nm using an ELISA plate reader (BioTek, Winooski, VT, USA).

##### Studies of Antifungal Activity

In this study, VCZ and L3NCMPs were tested to determine their in vitro antifungal activity against two standard fungal strains, *Aspergillus niger* (ATCC 16404) and *Aspergillus flavus* (ATCC 9197); all strains were cultured on potato dextrose agar (PDA) medium and potato dextrose broth (PDB) medium at 28 °C for 72 h. Their antifungal activities were evaluated using the agar-well diffusion assay previously described in [32], with some modifications. Briefly, standard strains were grown for 72 h in PDA (Oxoid) at 28 °C.

The strain suspensions were diluted in PDB and adjusted to 0.5 on the MacFarland scale at 530 nm using a spectrophotometer. Next, each microbial suspension was spread on the surface of the PDA (Oxoid) plates using a sterile cotton swab. The PDA plate surfaces were perforated with a sterile cork borer (6 mm). Then, 50–100 µL of VCZ suspension with a concentration range from 10 to 25 µg/mL and L3NCMPs were transferred into each well. The plates were incubated aerobically for 72 h at 28 °C. The inhibition zone’s diameter was measured around each well, and the results were recorded in mm as an average of three trials.

##### Stability Study

After preparing the spray-dried samples, they were added to glass vials and kept in a desiccator. The samples were stored for three months under different temperature and humidity conditions, i.e., at 25 °C with 60 ± 5% relative humidity (RH) and 40 °C with 75 ± 5% RH, as specified for DPIs by the USFDA stability guidelines. The spray-dried samples were visually inspected for any signs of discoloration and reassessed to determine their liposome particle size, PDI, zeta potential, and drug content after 0, 1, 2, and 3 months, as detailed in Sections Particle Size, Polydispersity Index, and Zeta-Potential Measurements and Encapsulation Efficiency.

#### 2.2.5. Statistical Data Analysis

The statistical differences between groups were analyzed via one-way ANOVA using GraphPad Prism 10.0.2 (GraphPad Software, Boston, MA, USA). *p* values ˂ 0.05 were chosen to denote statistical significance. The results are expressed as the mean ± standard deviation (*n* = 3).

## 3. Results

### 3.1. Physicochemical Characterization of VCZ-L Formulations

#### 3.1.1. Particle Size, Polydispersity Index, and Zeta-Potential Measurements

The liposomes were analyzed using DLS to determine their mean particle size, PDI, and zeta-potential values. The results presented in Table 1 show that the particle size of all liposome formulations was less than 100 nm, with PDI values in the range of (0.116–0.125 < 0.2). The zeta-potential values increased with increasing VCZ/lipid ratios, ranging from −2.74 mV for the blank to −26 mV for F 3 (1.5:1).

#### 3.1.2. Morphology of Liposomes

**Figure 1:**
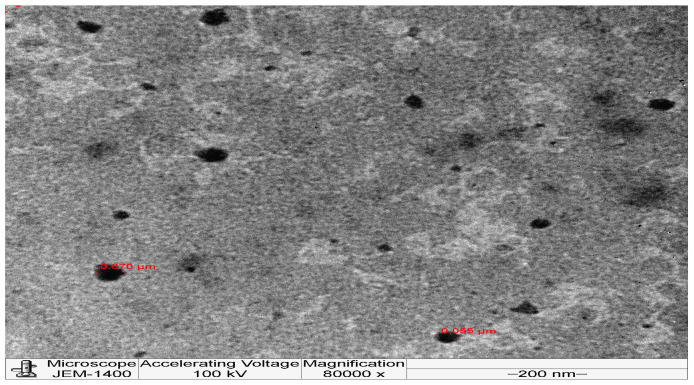
Transmission electron microscopy micrographs of F 3 (voriconazole/lipid ratio 1.5:1) at a magnification power of 80,000×.

Figure 1 presents the TEM image obtained for the selected VCZ-Ls formulation F 3. The liposomes exhibited a relatively uniform, spherical morphology and were evenly distributed in the microscopic field, with sizes in good agreement with the results obtained from the Nano zetasizer (˂100 nm).

#### 3.1.3. Encapsulation Efficiency %

**Figure 2:**
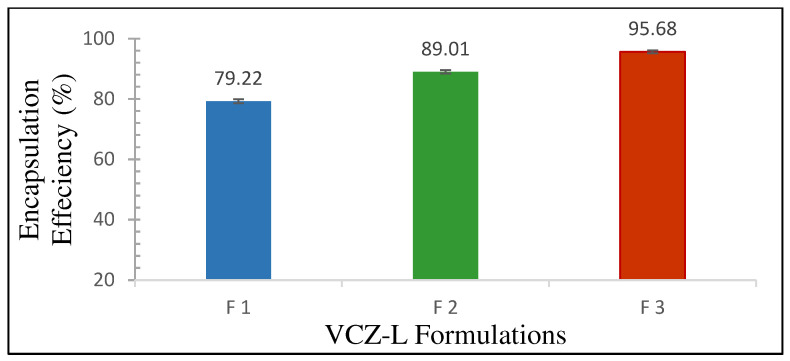
Encapsulation efficiency of voriconazole–liposomal formulations (voriconazole/lipid ratio); F1 (0.5:1), F 2 (1:1), and F 3 (1.5:1) (mean ± SD, *n* = 3).

As shown in Figure 2, the percentage of VCZ encapsulated in the liposomes increased as the VCZ/lipid ratio increased. The highest percentage of encapsulated VCZ (95.68 ± 0.47%) was obtained for F 3 (1.5:1).

#### 3.1.4. In Vitro Release Study

**Figure 3:**
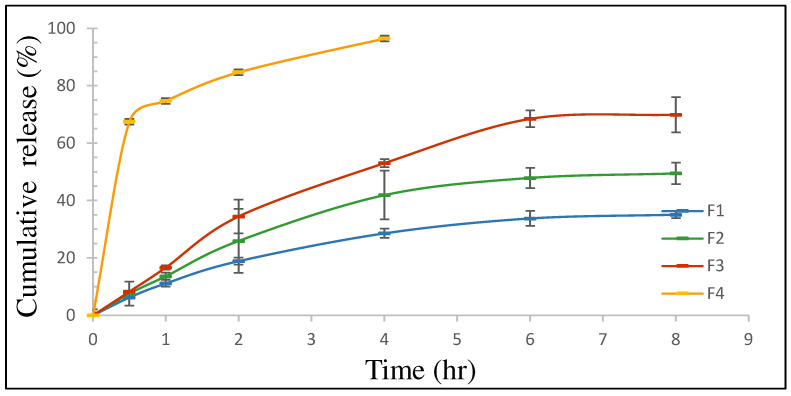
In vitro release profile of voriconazole in phosphate-buffered saline containing 0.2% Tween 80 at pH 7.4; F 1 (0.5:1), F 2 (1:1), F 3 (1.5:1), from voriconazole-loaded liposomes at different voriconazole/lipid ratios, and F 4 voriconazole suspensions (mean ± SD, *n* = 3).

The in vitro drug release of VCZ from different VCZ-Ls formulations was carried out in PBS solutions containing 0.2% Tween 80 at a neutral pH of 7.4 and a temperature of 37 °C, as presented in Figure 3. The drug release profile showed that 96.49 ± 2.50% of the VCZ was released from the F 4 (VCZ suspension) within four hours. On the other hand, the developed VCZ-Ls formulations demonstrated controlled and slow drug release over 8 h. The cumulative release% of VCZ reached 69.93 ± 2.91%, 49.51 ± 3.55%, and 35.08 ± 2.60% over 8 h for F 3, F 2, and F 1, respectively. Therefore, the highest-performing drug-release profile was observed for F 3, which contained the highest proportion of VCZ.

To gain a better understanding of the mechanism of the release of VCZ from the tested formulations at pH 7.4, the data were fitted to the zero-order, first-order, Higuchi, Korsmeyer–Peppas, and Hixson–Crowell models using DDSolver software; the results are presented in Table 2. The Korsmeyer–Peppas model showed the highest r^2^ value for all samples. Additionally, the values of *n* were 0.536, 0.567, and 0.629 for F 1, F 2, and F 3, respectively, indicating non-Fickian diffusion. Consequently, it was concluded that the drug release mechanism mainly comprised a combination of the diffusion and erosion of VCZ liposomes.

### 3.2. Characterization of Nanocomposite Microparticles (NCMPs)

#### 3.2.1. Production Yield %

Table 3 displays the powder yield values obtained after SD. The lowest mean yield was observed for L1NCMPs prepared with a lipid/LEU ratio of 1:0.5 *w*/*w* (66.80 ± 3.43%). On the other hand, the highest yield was noted for L3NCMPs prepared with a lipid/LEU ratio of 1:1.5 *w*/*w* (80.41 ± 4.52%). The production yield gradually increased as the proportion of the LEU ratio increased.

#### 3.2.2. Determination of the Drug Content of the Spray-Dried Powders

The drug content of the spray-dried powders was found to be in the range of 90.7 ± 6.28% to 93.5 ± 3.9% of the anticipated amount, demonstrating that there was no deleterious effect of the spray-drying process on the VCZ (Table 3).

#### 3.2.3. Scanning Electron Microscopy

**Figure 4:**
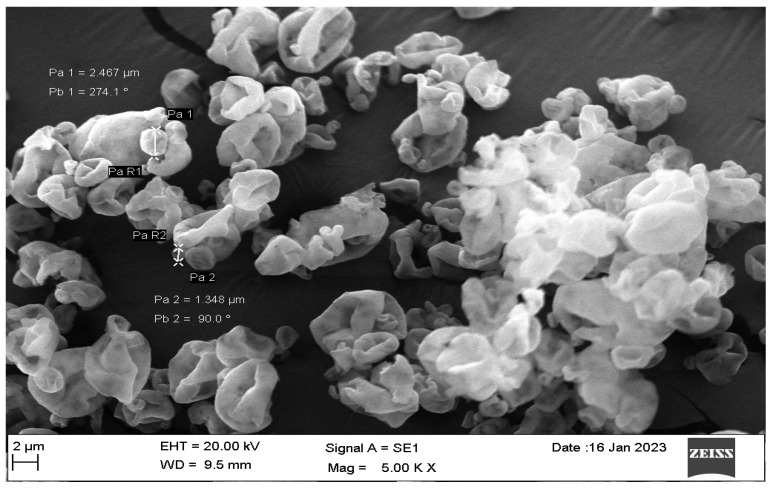
SEM images of the L3NCMP formulation prepared using a lipid/L-leucine ratio of 1:1.5, taken at a magnification 14,000 and 15.0 Kv.

The SEM micrographs of L3NCMPs in Figure 4 show that the particles were wrinkled and rough.

#### 3.2.4. Fourier Transform Infrared Spectroscopy

**Figure 5:**
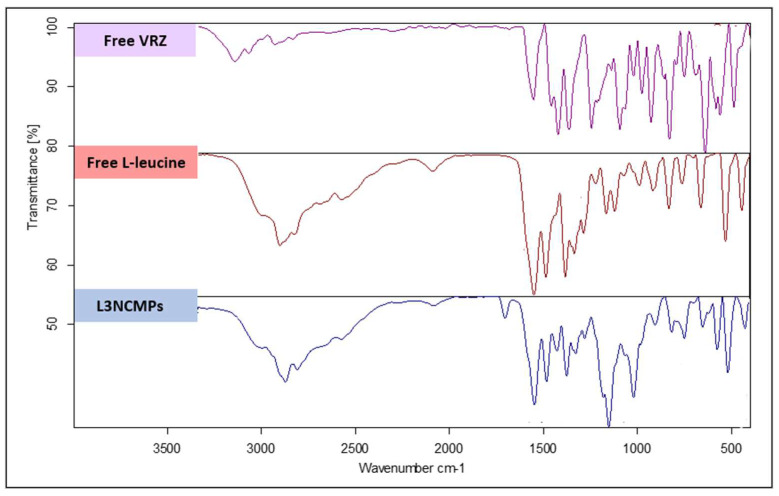
Fourier transform infrared spectra of spray-dried VCZ-loaded liposomes with L-leucine (L3NCMPs), compared to free voriconazole and L-leucine.

The FTIR spectrum obtained for VCZ, shown in Figure 5, displays an O-H stretching band at 3191.89 cm^−1^, while the bands at 1589.82–1457.26 cm^−1^ are related to the characteristic aryl C-N stretching. The observed spectral region between 1051.9 and 957.96 cm^−1^ represents stretching of the C-F group. The peak at 3119.35 cm^−1^ is attributed to the stretching vibrations of aromatic rings. The bands at 2978.19–2345.17 cm^−1^ are attributed to C=C aromatic stretching, while the bands between 2062.53 and 1718.70 cm^−1^ are attributed to alkane CH stretching. The peaks between 886.88 and 668.30 cm^−1^ are related to the bending of the aromatic =C-H group.

The FTIR spectrum of pure LEU shown in Figure 5 demonstrates two peaks, at 1507.03 and 1572.34 cm^−1^, which can be attributed to the stretching of carbonyl (C=O) groups on the amino acid. The appearance of these carbonyl peaks at low wavenumbers indicates the formation of hydrogen bonds between the carbonyl oxygen and the amine group. The peak observed at 2950.05 cm^−1^ is related to the vibration of LEU methyl groups.

The FTIR spectrum of L3NCMPs shown in Figure 5 is free from the characteristic peaks of VCZ, suggesting that the drug was fully incorporated into the spray-dried mixture. When VCZ was incorporated with LEU, the C-N stretching observed at 1589.82–1457.26 cm^−1^ shifted to 1577.09–1455.22 cm^−1^. Similarly, the C-F stretching recorded at 1051.9–957.96 cm−1 shifted to 1041.49–926.55 cm^−1^. Additionally, the C=C aromatic stretching detected at 2978.19–2345.17 cm^−1^ shifted to 2619.33–2122.55 cm^−1^. These shifts could be due to the molecular interaction of the aromatic moieties of VCZ and LEU during the SD process. In addition, The FTIR spectrum obtained for the spray-dried formulation (L3NCMPs) showed a shift in the bands of LEU from 1507.03 cm^−1^ and 1572.34 cm^−1^ to 1511.95 cm^−1^ and 1577.09 cm^−1^, respectively. Additionally, the peak of the LEU methyl groups at 2950.05 cm^−1^ shifted to 2922.16 cm^−1^. Again, these shifts suggest the possible formation of a new intramolecular hydrogen bond interaction during the spray dying of VCZ with LEU.

#### 3.2.5. Cell Viability Study

**Figure 6:**
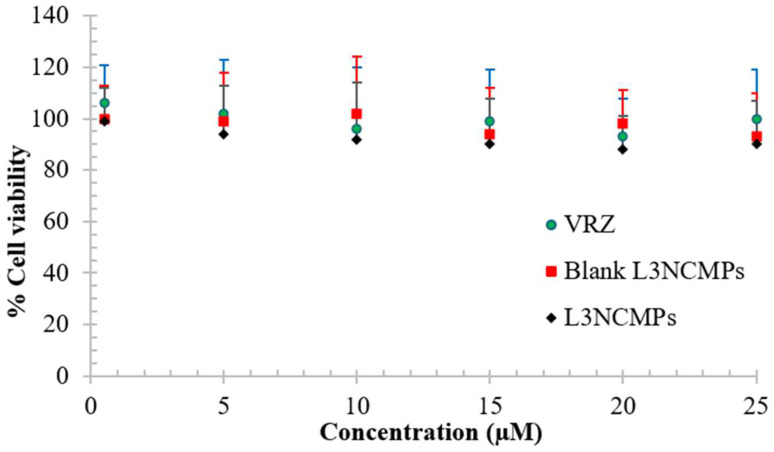
Cell viability assay of voriconazole, L3NCMPs (lipid/L-leucine ratio of 1:1.5), and blank L3NCMPs (lipid/L-leucine ratio of 1:1.5) on A549 cells for 72 h employing MTT assay (mean ± SD, *n* = 3).

Neither the blank L3NCMP nor the L3NCMP powder affected cell viability, as more than 85% of cells were found to be viable compared to VCZ (*p* > 0.05) when treated with the highest drug concentration (Figure 6).

#### 3.2.6. Studies of Antifungal Activity

In this study, the antifungal activities of the L3NCMP formulation and different concentrations of VCZ, ranging from 10 to 25 µg/mL, were examined against both *Aspergillus niger* (ATCC 16404) and *Aspergillus flavus* (ATCC 9197), using the agar-well diffusion method. The results were obtained by measuring the inhibition zone (clear zone) around each well in (mm).

The data in Table 4 demonstrate the antifungal activity of L3NCMPs compared to pure VCZ, with the latter exhibiting potent antifungal activity against the tested Aspergillus spp., and a slightly greater effect on *Aspergillus niger*. On the other hand, the maximum antifungal activity was observed with 2.5 µg/mL L3NCMPs, against *Aspergillus niger*, as indicated by the fact that it had the largest zone of inhibition of 40 mm.

**Figure 7:**
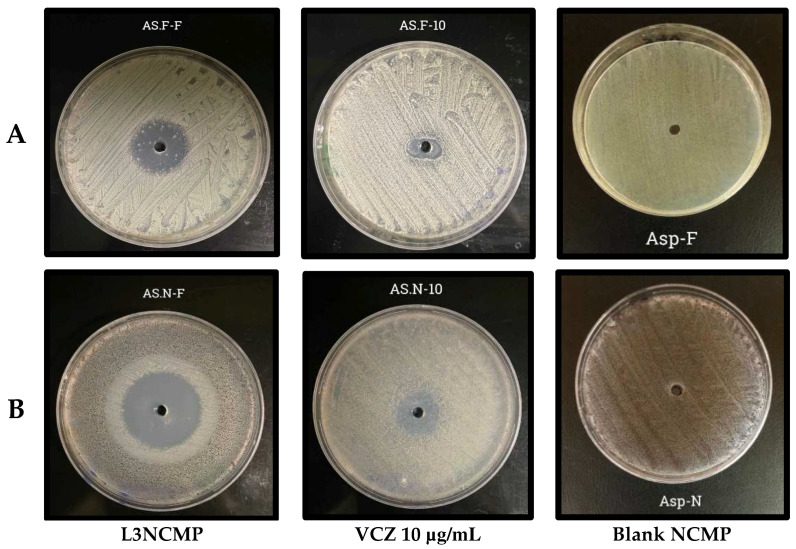
The inhibition zone of L3NCMPs (lipid/L-leucine ratio of 1:1.5) and voriconazole (VCZ) solution (10 µg/mL) against (**A**) *Aspergillus flavus* and (**B**) *Aspergillus niger*.

The results shown in Figure 7 reveal that L3NCMPs were more potent than VCZ alone against both *Aspergillus niger* (ATCC 16404) and *Aspergillus flavus* (ATCC 9197), with clear zones of inhibition of 40 mm and 30 mm, respectively, and with no effect of blank liposomes. Thus, the L3NCMPs were more effective than the reference drug. The minimal inhibitory concentration (MIC) of L3NCMPs appeared to be lower compared to the drug alone, at 7 μg/mL Furthermore, L3NCMPs were able to inhibit growth at sub-MIC levels with slightly higher efficiency.

#### 3.2.7. Stability Study

Table 5 demonstrates the stability data of L3NCMPs under different storage conditions. The L3NCMP formulation exhibited no significant change (*p* > 0.05) in particle size, PDI, or drug content when stored at room temperature for three months. Initially, after two months of storage, no significant increase was observed in the size of the liposomes, PDI, and drug content at 40 ± 2 °C/75 ± 5% RH. However, after three months, a significant increase in particle size (105 ± 32.43) and a ~20% reduction in drug content were observed (*p* < 0.05).

## 4. Discussion

In this study, three lipid components were used for liposome formulation, namely, DSPC, DOPC, and DOPE. DSPC was chosen as a zwitterionic phosphatidylcholine with long and linear acyl chains, which are also saturated and even-chained. The phospholipids increase stability against chemical degradation, leading to a reduction in drug leakage from liposomes during storage and in vivo transit). DOPC was selected to increase the fluidity of the liposomal membrane, due to its high fluidity at room temperature. On the other hand, DSPC keeps the liposomal membrane in the gel phase, with a Tm of +55 °C [33]. Additionally, DOPE is a non-bilayer-forming lipid that can efficiently modulate the fusion potential of liposomes. These lipids, which have different chain lengths and degrees of saturation, can be used to adjust the membrane’s dynamics and phase properties as needed [34].

The film hydration method was utilized in this study to encapsulate VCZ into liposomes with high efficiency and small vesicle sizes (<100 nm) [35]. The obtained liposomes showed polydispersity index values of less than 0.2, which indicated a narrow size-distribution. These results are consistent with those of Mayer et al., who demonstrated that similar procedures could produce uniformly sized liposomes by employing filters with pore sizes ranging from 30 to 400 nm [36]. The small size of these liposomes allows them to penetrate cell membranes and be taken up by the cells, resulting in efficient drug accumulation at the target site [36].

Depending on the lipid composition, liposomes and lipid NPs can have a negative, neutral, or positive net charge. Liposomes that lack a surface charge (neutral liposomes) tend to aggregate more easily, which can reduce their physical stability [37]. The higher zeta-potential values of the obtained liposomes (Table 1) suggest that they have greater potential for physical stability.

The TEM analysis of VCZ-Ls showed they are relatively uniform and homogenous and have a spherical shape with a smooth surface (Figure 1).

The highest EE % of VCZ, 95.68% ± 0.47%, was obtained at a 1.5:1 VCZ/lipid ratio with good reproducibility (Figure 2). VCZ is a lipophilic substance that is compatible with the phospholipids utilized in liposome preparation and becomes a part of the bilayer. The high EE % also indicates that the lipid bilayer could significantly solubilize the hydrophobic drug, allowing it to be transported through the phospholipid bilayer. This could be an effective solution for the low oral bioavailability of VCZ. Additionally, Nallamothu et al. found that as the drug/lipid ratio increased from 1:10 to 2:10, the total drug content in the liposome formulation also increased [38]. However, when the drug/lipid ratio was further increased to 4:10, the liposome formulation’s total drug content did not increase. Instead, the quantity of free drugs increased significantly, leading to a decrease in the EE % of entrapped drugs. This indicates that at higher drug/lipid ratios, there might not be enough lipids to entrap the drug, and most of the drug may exist in a free or un-entrapped form [38].

PBS was chosen as the dissolution medium because of its simple composition, and its ability to show sustained release or pH-independent characteristics. The pH of PBS was set to 7.4 to match the pH of the lung lining fluid [39]. All the liposomal formulations of VCZ exhibited a controlled drug release profile for 8 h. The highest-performing drug-release profile was observed in F 3 (1.5:1), which contained the highest drug proportion of VCZ, reaching 69.93% ± 2.91% over 8 h, as shown in Figure 3. These results indicate that the liposomes demonstrated release profiles which were significantly more controlled, and the VCZ was effectively loaded. Mathematical models are important tools for designing pharmaceutical formulations, evaluating drug release processes in vitro and in vivo, and finding optimal designs for new systems. It has been stated that the release of drugs from liposomes can be explained by three different mechanisms: diffusion, erosion, and diffusion–erosion [40]. This study found that the release profiles of VCZ-Ls were supported by the Korsmeyer–Peppas model at pH 7.4, and, as indicated by this model, had the highest r^2^ value. Additionally, the *n* values of the release exponent were estimated to be 0.536, 0.567, and 0.629 for formulations prepared with ratios of 0.5:1, 1:1, and 1.5:1, respectively, indicating non-Fickian diffusion kinetics (0.5 ˂ *n* ˂ 1) [40]. Therefore, the drug release mechanism could be conclusively attributed to a combination of the diffusion and the erosion of liposomes that contained VCZ (Table 2).

Since the VCZ-L F 3 exhibited an average size of 55 ± 3.46 nm, it cannot be directly used for inhalation. Most of the inhaled dose will be exhaled, with only a minimal amount of the dose deposited in the lungs [41]. Furthermore, there are specific problems with stability when utilizing only liposome dispersion [42]. Therefore, F 3 was formulated using SD to create NCMPs using LEU as a carrier and dispersibility enhancer to enable its administration using a DPI. The size of the particles was in the respirable range, ranging from 3.65 µm to 4.09 µm, with a high production yield% (66.80 ± 2.5%–80.41 ± 5.29%) obtained for all formulations (Table 3). The SEM pictures (Figure 4) revealed a wrinkled and rough surface resulting from a vapor pressure build-up due to water evaporation during the SD process. This phenomenon typically occurs with hydrophobic amino acids such as LEU [43]. This observation agrees with many studies that have found irregular surfaces in NCMP prepared with LEU and also found that increasing the ratio of LEU resulted in a higher yield value [44,45]. This study chose L3NCMP prepared with a lipid/LEU ratio of 1:1.5 *w*/*w* for additional studies, as it had the highest percentage yield.

The FTIR studies showed either no interactions or weak interactions, eliminating incompatibility between VCZ and the chosen excipients (Figure 5).

The safety of the formulation and its compatibility with lung cells are crucial factors in developing a PDDS. Inhaling any toxic ingredients may reduce lung surfactant and the recruitment of phagocytizing cells in the lower respiratory tract, which can be harmful [46]. Therefore, the MTT assay was used to study the safety of the optimized formulation, as it is a widely accepted and standardized method for evaluating the cytotoxic effects of drugs and formulations against fungal cells. The MTT assay provides a quantitative measure of the antifungal potency of the VCZ formulations, which is a crucial consideration for ensuring the safety and efficacy of the PDDS [47]. Our in vitro viability studies revealed the safety of the L3NCMPs in bronchoalveolar epithelial cells at the highest concentration tested, as shown in Figure 6. These findings suggest that VCZ can be safely administered to the lungs. The results of these in vitro viability studies are consistent with toxicity studies of inhaled VCZ solution in rats conducted by Tolman and colleagues [48], as well as in vitro VCZ A495 cytotoxicity studies carried out by Kaur et al. [49].

The effects of the L3NCMP formulation and the pure VCZ against Aspergillus strains were studied using the agar-well diffusion method. This technique is widely accepted and commonly used in mycological research and clinical diagnostics to assess the effects of various antifungal agents against fungal organisms, including Aspergillus strains. This qualitative method allows for the visual observation and measurement of the zone of inhibition, which provides an indicator of the diffusion and antimicrobial effectiveness of the VCZ formulations against the target fungal strains [50]. Overall, the results in Figure 7 show that the zone of inhibition was higher for the L3NCMP formulation than the pure VCZ. This is in agreement with Horvat and colleagues’ study, which suggested that NPs interacted better with the fungal cell wall than did the reference drugs, enhancing drug efficacy [51]. Further studies are required to evaluate the efficiency of these characteristics in combatting infections and diseases in practical scenarios.

Liposomal formulations carrying drugs may become destabilized over time due to exposure to humidity and unsuitable temperatures. Storage can cause physicochemical changes, such as liposomal aggregation, fusion, and the loss of drugs. These changes can significantly impact the formulation’s in vivo performance [52]. Additionally, phospholipids may undergo hydrolysis reactions, forming fatty acids and lysophospholipids. The oxidation of lipids may cause structural integrity problems and lead to the release of trapped drugs [53]. Therefore, short stability studies of the L3NCMP formulation were conducted to assess such changes. The liposome size was measured after the rehydration of the spray-dried powders. The study results indicated no significant change (*p* > 0.05) in the size of the liposomes, PDI, or VCZ content when stored at room temperature for 3 months (Table 5). This suggests that the formulation is chemically stable under these storage conditions. The zeta potentials of the reconstituted liposome dispersions at different storage times were constant, regardless of size. On the other hand, exposure to high temperature and humidity resulted in a 20% reduction in the VCZ content in powders, highlighting the importance of protection from high humidity and temperature for optimum formulation performance. A similar finding was also reported by Arora and colleagues, in which a highly respirable inhalable dry-powder formulation of VCZ and leucine exhibited storage stability under room-temperature conditions for up to 3 months, potentially reducing the need for cold-chain storage of the dry powders [54]. Additionally, studies have shown that adding leucine to antibiotic formulations prevented the pronounced crystallization of antibiotics after long-term storage by reducing moisture ingress and improving the developed formulations’ aerosolization performance [55,56]. In summary, further research is required to determine the drug instability’s root cause, whether it be hydrolysis or other moisture-mediated pathways; this is an important next step. And evaluating the packaging material’s moisture vapor transmission rate could help guide the selection of more suitable container closure systems.

## 5. Conclusions

This study developed inhalable VCZ liposomes for treating IPA. Using a thin-film hydration method, the researchers successfully incorporated VCZ into liposomes with different VCZ-to-lipid ratios. The resulting VCZ liposomes demonstrated good physical stability and sustained drug release properties at pH 7.4. The liposomal formulation prepared with a VCZ/lipid ratio of 1.5:1 was selected for further investigation of its antifungal activity as a dry-powder inhaler. The researchers successfully prepared inhalable VCZ dry powder using SD with LEU; this demonstrated desirable lung delivery properties, including physiochemical characteristics, a good safety profile, and antifungal activity. Overall, the results of this study support the use of liposomal NPs as a promising drug delivery system for VCZ, a DPI with high antifungal activity for the treatment of IPA.

## Figures and Tables

**Table 1:** Mean particle size, PDI, and zeta-potential values of voriconazole liposomal formulations (mean ± SD, *n* = 3).

Formulations (VCZ/Lipid Ratio)	Mean Particle Size (nm)	PDI	Zeta Potential (mV)
F 1 (0.5:1)	47.87 ± 1.47	0.123	−19.4 ± 3.98
F 2 (1:1)	53.44 ± 0.79	0.117	−23.4 ± 5.39
F 3 (1.5:1)	53.46 ± 0.93	0.116	−26.0 ± 4.06
Blank	43.27 ± 1.62	0.125	−2.74 ± 4.67

**Table 2:** Modeling of voriconazole release kinetics from different liposomal formulations at pH = 7.4.

Model/8 h	Parameter	F 1	F 2	F 3
Zero-order	r^2^	0.6861	0.7334775	0.8315
	k_o_ (h^−1^)	5.360	7.592	10.495
First-order	r^2^	0.8099	0.8918	0.9604
	k_1_ (h^−1^)	0.068	0.110	0.182
Higuchi	r^2^	0.9648	0.942858	0.9362
	k_1_ (h^−½^)	13.094	18.462	25.256
Korsmeyer–Peppas	r^2^	0.9677	0.9519	0.9836
	k_KP_ (h^−n^)	12.369	16.587	20.526
	“n” value	0.536	0.567	0.629
Hixson–Crowell	r^2^	0.7728	0.8492	0.9548
	kHC	0.21	0.32	0.51

**Table 3:** The geometric particle size, yield %, and drug content of NCMPs prepared through the spray drying of F 3 (voriconazole/lipid ratio 1.5:1) using different ratios of L-leucine. (Mean ± S.D, *n* = 3).

	L1NCMPs	L2NCMPs	L3NCMPs
Ratio (lipid/LEU)	1:0.5	1:1	1:1.5
Geometric particle size (µm)	4.09 ± 0.19	3.74 ± 0.14	3.65 ± 0.73
Production yield (%)	66.80 ± 2.5	72.63 ± 4.13	80.41 ± 5.29
Drug content %	93.5 ± 3.9	90.7 ± 6.28	91.15 ± 4.7

**Table 4:** The inhibitory effect of L3NCMPs (lipid/L-leucine ratio of 1:1.5) versus voriconazole, against *Aspergillus niger* ATCC 16404 and *Aspergillus flavus* ATCC 9197 (mean ± SD, *n* = 3).

Fungal Strains	L3NCMPs (µg/mL)	Voriconazole (µg/mL)	
	2.5	10	25
	Zone of Inhibition (mm)		
*Aspergillus flavus* ATCC 9197	30	14	22
*Aspergillus niger* ATCC 16404	40	20	26

**Table 5:** Stability study data for L3NCMPs (lipid/L-leucine ratio of 1:1.5) after storage at room temperature and under accelerated conditions for 3 months.

Time	Storage Condition (Temperature and Humidity)	Parameters		
		Particle Size (nm)	PDI	Drug Content (%)
Zero Time		55 ± 3.46	0.145 ± 0.13	91.15 ± 4.7
After 1st month	25 ± 2 °C/60 ± 5% RH	53 ± 5.34	0.163 ± 0.12	90.95 ± 6.28
	40 ± 2 °C/75 ± 5% RH	58 ± 7.48	0.129 ± 0.12	90.42 ± 3.75
After 2nd month	25 ± 2 °C/60 ± 5% RH	60 ± 8.91	0.194 ± 0.15	91.15 ± 7.42
	40 ± 2 °C/75 ± 5% RH	67 ± 13.57	0.279 ± 0.17	89.74 ± 9.21
After 3rd month	25 ± 2 °C/60 ± 5% RH	86 ± 21.75	0.284 ± 0.21	83.15 ± 5.7
	40 ± 2 °C/75 ± 5% RH	105 ± 32.43	0.322 ± 0.24	72.86 ± 4.59

## Data Availability

The data is contained in the manuscript.
